# Normal twin PET: personalized generative modeling for confounder correction and anomaly detection in whole-body PET/CT

**DOI:** 10.1038/s41598-025-26827-y

**Published:** 2025-11-28

**Authors:** Christian Hinge, Anders Bertil Rodell, Sven Zuehlsdorff, Bruce Spottiswoode, Kirsten Korsholm, Barbara Malene Fischer, Claes Nøhr Ladefoged, Flemming Littrup Andersen

**Affiliations:** 1 Department of Clinical Physiology and Nuclear Medicine, Rigshospitalet, Copenhagen, Denmark; 2 Siemens Healthcare A/S Denmark, Ballerup, Denmark; 3 Siemens Medical Solutions USA, Inc., Knoxville, TN USA; 4 Department of Applied Mathematics and Computer Science, Technical University of Denmark, Kongens Lyngby, Denmark; 5 Department of Clinical Medicine, Copenhagen University, Copenhagen, Denmark

**Keywords:** Artificial intelligence, Anomaly detection, LAFOV, FDG-PET, Digital twin, Confounder correction, Cancer, Computational biology and bioinformatics, Medical research, Oncology

## Abstract

Variable physiological [^18^F]FDG uptake patterns and a lack of labelled data make it challenging to automatically distinguish normal from pathological suspicious uptake in whole-body PET/CT imaging. We propose a deep learning method that generates patient-specific normal twin PET images to serve as personalized references for quantitative analysis and unsupervised detection of pathological anomalies. We developed an image-to-image generative model that synthesizes normal reference twin PET (ntPET) images from CT scans, patient demographics, and acquisition parameters. The model was trained on 2,538 pseudo-normal PET/CT studies, including stable lymphoma patients and manually disease-masked clinical scans. Model performance was evaluated on 177 test studies achieving 89.3% explained variance and 18.0% mean absolute relative error. We introduced a novel “twin correction” method which reduced SUV_mean_ variance by up to 90% in various organs and successfully reduced confounding normally occurring effects of patient sex, age, fat mass, and uptake time. Finally, anomaly detection and unsupervised tumor segmentation was achieved by comparing actual PET scans with their normal twins. The ntPET-based method achieved a dice score of 49.3% on the AutoPET dataset without requiring tumor annotations for training. In conclusion, the proposed ntPET methodology employs personalized normal references to achieve disease-agnostic patient-specific analysis of PET images.

**Supplementary Information:**

The online version contains supplementary material available at 10.1038/s41598-025-26827-y.

## Introduction

Combined Positron Emission Tomography/Computed Tomography (PET/CT) is an invaluable tool for the diagnosis and treatment assessment of patients with pathology^1–3^. PET imaging using 2-[^18^F]fluoro-2-deoxy-d-glucose ([^18^F]FDG) radiopharmaceutical depicts glucose metabolism while CT provides anatomical context. However, analyzing whole-body PET/CT images remains a labor intensive process.

Artificial Intelligence (AI) has been investigated extensively for the automatic analysis of [^18^F]FDG-PET-images, but its translation to clinical practice is often limited by poor explainability and generalization to clinical populations^4–6^. Furthermore, supervised classification and segmentation models often need to be trained or finetuned anew for each disease, and for some diseases, it can be expensive or impossible to obtain large, labelled datasets. One confounding factor in whole body 3D imaging is that the disease-affected areas often comprise a small fraction of the total image. The bulk of the PET-image, albeit being of little relevance to the investigated disease, contains significant interpatient variations and physiological uptake patterns, which can lead to model failures if not adequately represented in the training data^7,8^. Consequently, there has been a desire for more transparent and disease agnostic analysis methods that rely less on large, labelled datasets.

Unsupervised anomaly detection (UAD) is a promising technique which utilizes large unlabeled datasets of normal controls to create a distribution of normal PET images^9–11^. Disease, inflammation and other suspicious uptake, which we will refer to as “anomalies”, can then be quantified as areas of deviation between the observed PET image and the estimated “normal” distribution of predictable occurring PET uptake. UAD is used for semi-quantitative analysis in brain PET/MRI investigations, where patient scans are compared against an atlas of negative controls, i.e., a distribution of normal brains^12^. However, since such normal atlases reflect population-level means and variances, the comparison may fail to account for uptake patterns induced by unique brain anatomy and other patient-specific characteristics^13^. Addressing this, some UAD methods employ AI autoencoder or image-translational models to create a normal PET atlas tailored to the patient’s brain. Since such an AI-derived atlas depicts a hypothetical normal version of the patient’s PET image, we refer to it as a normal twin PET (ntPET) image^11,13^.

Extending the ntPET approach from brain to whole-body imaging remains challenging, firstly, due to a lack of negative controls studies – especially ones acquired on the newer long-axial field-of-view (LAFOV) scanners^14^. Furthermore, whole body PET images exhibit both high anatomical and physiological variations caused by patient specific effects such as weight, ambient temperature, gender, fasting state, rest, menstrual cycle, etc^15,16^. Ultimately, this can make it difficult to estimate a normal twin image and, by extension, distinguish normal from suspicious uptake patterns^7,17^.

Addressing these challenges, we present a whole-body ntPET model, which generates normal value PET images tailored to the patient anatomy, demographics, and acquisition parameters. We then demonstrate how the ntPET can be used to remove the predictable confounding effects of sex, season, and fat body mass on SUV_mean_ measurements. Secondly, we show that a straight-forward disease masking procedure enables incorporating a large cohort of non-control studies in the training data and still obtain normal appearing ntPET images. Finally, the ntPET model is demonstrated for unsupervised tumor segmentation.

## Materials and methods

**Fig. 1:**
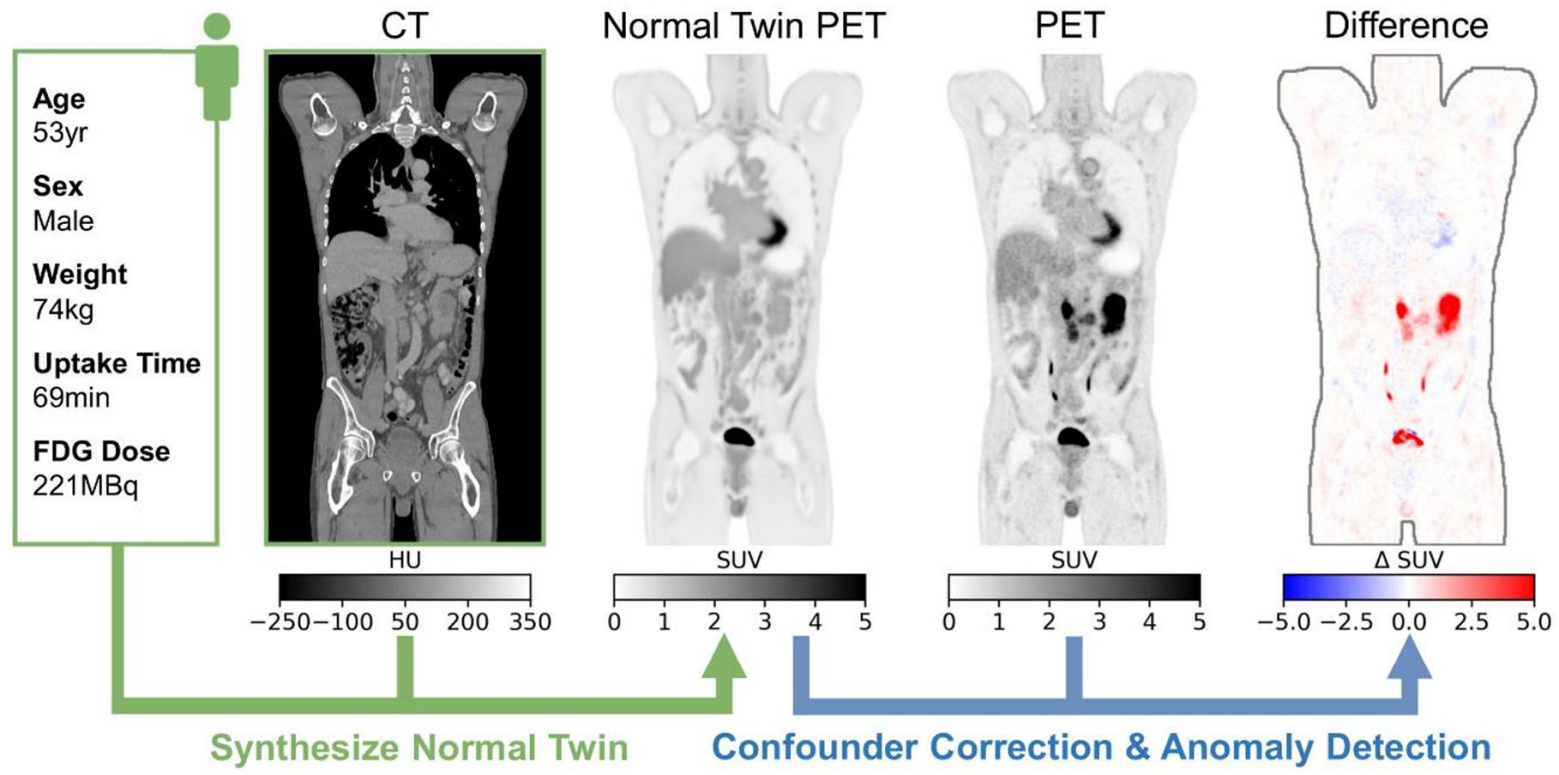
Normal Twin PET (ntPET) pipeline. The model generates normal twin PET images from CT and patient attributes. Comparison with the true PET enables personalized confounder correction and unsupervised anomaly detection (shown: DLBCL patient with tumor and physiological bladder uptake).

The normal twin approach consists of two steps (Fig. 1):

1. Synthesize normal twin PET: A normal twin PET image is generated using the patient’s CT, patient demographics, and acquisition parameters.
2. Confounder correction and anomaly detection: Confounder-corrected organ SUV-means are calculated, and suspicious uptake patterns are identified both by comparing the actual PET with the normal twin PET.

### Data

A common approach for synthesizing a normal twin PET image would involve training a deep generative model on a large and diverse cohort of normal PET control studies, where we define normality as an absence of clinically significant PET findings. Given the scarcity of such datasets, we instead employ a large, multi-site, multi-scanner cohort of ‘pseudo-normal’ [^18^F]FDG PET/CT studies from three datasets. This pseudo-normal cohort includes patients without clinically significant FDG-avid findings, such as post-treatment stable cases, but also active disease cases with disease regions masked during training:

- Stable lymphoma: 1475 studies of “stable” lymphoma patients acquired at Rigshospitalet, with “stable” defined by Deauville score ≤ 3 or complete remission at follow-up^18^. Data was acquired on a LAFOV PET/CT (*n* = 177) or a short-axis FOV PET/CT (*n* = 1,298).
- Masked LAFOV: 713 LAFOV studies from the clinical cohort of patients referred to Rigshospitalet primarily due to cancer or inflammation. Areas suspicious of disease were masked out during model training (see Sect. “Disease Masking”).
- AutoPET control: 471 negative control studies from the public AutoPET dataset^19^.

This cohort represents not a population of healthy humans, but a population of patients largely void of clinically significant disease, which is in line with the chosen definition of normality. For the unsupervised anomaly detection, ntPET images were synthesized and compared to the true PET images of patients with active cancer from the following two datasets:

- Active lymphoma: 121 LAFOV studies of “active” lymphoma patients acquired at Rigshospitalet, which includes patients with Deauville score 4 or 5, patients with disease recurrence, and patients in staging.
- AutoPET cancer: 501 studies of patients with malignant melanoma, lymphoma, or lung cancer from the AutoPET I dataset^19^.

All patient-specific data acquired at Rigshospitalet (Copenhagen, Denmark) were handled in compliance with the Danish Data Protection Agency Act no. 502, including full anonymization. The use of retrospective patient data without informed participant consent was approved by the Danish National Committee on Health Research Ethics (NVK) (ref. 2213953).

#### Splits

For the pseudo-normal cohort, two test splits were made comprising 25% of the AutoPET control studies (*n* = 121), and all LAFOV studies of the Stable Lymphoma dataset (*n* = 177), respectively (Table 1). The remaining 2538 studies were used for training and validation of the ntPET model.

The Active Lymphoma and AutoPET Cancer datasets were used exclusively for final testing of the unsupervised anomaly detection (Table 1). All splits were performed on subject-level to prevent data-leakage.

**Table 1:** Data overview. The pseudo-normal cohort (*n* = 2659) was used for training, validating, and testing the ntPET model. The cancer cohort (*n* = 621) was used for testing unsupervised anomaly detection. Values: mean ± standard deviation.

Cohort	Source	Dataset	Split	*N*	Gender [M/F]	Age [Years]	BMI [kg/m^2^]
*Pseudo-normal* (*n* = 2659)	*Rigs.*	*Stable lymphoma*	*Train/val*	*1298*	*754/546*	*60.5 ± 17.3*	*24.9 ± 4.4*
			*Test*	*177*	*102/75*	*58.8 ± 19.5*	*25.5 ± 4.8*
		*Masked LAFOV*	*Train/val*	*713*	*253/460*	*58.6 ± 15.6*	*24.7 ± 5.0*
	*AutoPET*	*AutoPET* *control*	*Train/val*	*380*	*205/173*	*58.9 ± 15.8*	*26.9 ± 7.8*
			*Test*	*91*	*50/41*	*60.0 ± 14.2*	*27.3 ± 6.1*
*Cancer* (*n* = 621)	*Rigs.*	*Active lymphoma*	*Test*	*121*	*58/63*	*66.0 ± 15.8*	*26.2 ± 6.5*
	*AutoPET*	*AutoPET* *cancer*	*Test*	*501*	*294/207*	*60.1 ± 16.5*	*26.7 ± 5.4*

#### Acquisition parameters

The Stable Lymphoma, Active Lymphoma and Masked LAFOV datasets, totaling 2309 whole-body PET/CT studies, were acquired retrospectively at Rigshospitalet, Copenhagen University Hospital. All scans were performed between September 2017 and November 2023 on a mix of clinical scanners; Biograph Vision Quadra (44%), Biograph Vision 600 (17%), Biograph mCT Flow (33%), and Biograph Truepoint (6%) (Siemens Healthineers). Contrast enhancing CT agents were used in 81% of the studies, and most scans covered mid-thigh to the base of the skull, with some scans extending to the lower extremities and top of the head. [^18^F]FDG PET acquisition parameters included an average of injected activity of 256 MBq (66 std) and an uptake time of 63 min ([57–65 min interquartile range]). Images were reconstructed to a median matrix size 440 × 440 mm^2^ using 3D ordinary poison - ordered subset expectation maximization (3D-OP-OSEM), point spread function (PSF) and a 2.0 mm (66%) or 2.5 mm (34%) gaussian filter.

The stable and active AutoPET datasets were acquired from two large medical centers in Germany on a variety of PET scanners (please see^19^ for details).

#### Preprocessing

PET/CT images were resampled to 2 × 2 × 2 mm^3^ and cropped to the clinical transaxial field of view (FOV) of the CT scanner, resulting in image dimensions 256 × 256 × Z, where Z denotes the axial dimension. PET images were normalized to standardized uptake value (SUV), and CT images were clamped to [−500, 600] Hounsfield units (HU) and rescaled to the interval [0, 2]^20^.

### Disease masking

Due to the scarcity of normal control LAFOV studies, we created the masked LAFOV dataset by randomly selecting 1,000 PET/CT studies from patients referred for [^18^F]FDG PET/CT on a LAFOV scanner between September 2021 and November 2023. To limit disease inclusion in the pseudo-normal training cohort, a nuclear medicine physician inspected coronal and sagittal PET MIP images (Supplementary Figure S1), which saw 287 studies excluded due to suspicion of extensive malignant or inflammatory pathology. For the remaining 713 studies, coarse disease masks were created by manually delineating malignant and inflammatory suspicious areas on the MIP images and expanding them along the anteroposterior and lateromedial directions (Supplementary Figure S1). During model optimization, loss within these masks was ignored, effectively training the ntPET model primarily on non-malignant regions.

### Models

The base ntPET model comprised a standard patch-based 3D UNet that used CT patches of size 256 × 256 × 32 as input and delivered corresponding PET patches^21^. During training, PET and CT patches were sampled randomly from the pseudo-normal cohort. At inference, patches were extracted with 66.6% axial overlap and averaged to obtain full ntPET volumes. The averaging was weighted by a generalized normal distribution to remediate uncertain predictions near patch-borders. Model variants with additional inputs (patient attributes, synthetic CTs, and organ segmentations) were investigated and are detailed below.

#### Study attributes

The anatomical information within the limited patch field of view (FOV) could fail to capture the global effects of covariates like age, sex, and uptake time. Addressing this, study attributes, i.e., patient demographics and acquisition parameters, were concatenated and encoded to a feature vector of length 16 using a 3-layer feed forward neural network (Fig. 1). This vector was added to the first 16 first channels of the bottom-most UNet layer by broadcasting across the spatial dimensions. The attributes included sex, age, and weight, [^18^F]FDG activity, scanner model, uptake time, scan time of the day, and scan day of the year. Discrete and time-dependent attributes were encoded using 3 dimensional embeddings and sin-cos transformations, respectively.

#### Synthetic CT

The localization of structures in the PET image was inferred solely from the CT image, hence misalignments of the two modalities could negatively affect the UAD. To reduce ntPET prediction artifacts in areas of common PET/CT misalignment, such as the liver and heart, AI-based “synthetic CT” (sCT) images were derived from a separately trained model and included in the UNet input. The sCT model comprised a 3D UNet identical to the ntPET model, but with the PET image as input and the CT image as output. The sCTs were informed by and therefore anatomically aligned to the PET images, hence their inclusion in the UNet model input was theorized to reduce misalignment artifacts in the final ntPET.

#### Segmentations

Organ segmentations including muscle, fat, bones and internal organs were derived using TotalSegmentator^22^, and concatenated to the model input using a 4-dimensional embedding layer. This was theorized to aid the model in identifying anatomical structures in the limited patch FOV.

#### Training scheme

Each model variant was trained on the pseudo-normal cohort (Table 1) for 80.000 steps in parallel on 4 NVIDIA A40 graphic card, using L1 loss and a batch size of 2. ADAM optimization was used with learning rate of 1e-04, which was decayed to 1e-05 at step 50.000. The loss ignored voxels found outside the patient body or inside the exclusion masks as described in Sect. “Disease Masking”.

#### Reference method

A simplistic constant organ map was created to serve as a base of comparison with the ntPET models. Using the Totalsegmentator masks, per-organ SUVmean values were obtained for the pseudo-normal training studies. These were then averaged to obtain a single pseudo-normal SUV_mean_ value per organ. Totalsegmentator organ masks of test studies were filled with these average values to create a pseudo-normal constant organ map that roughly conformed to the patient’s anatomy.

### Evaluation of ntPET

The performance of the ntPET models were assessed based on how accurately they could replicate pseudo-normal PET images from the Stable Lymphoma and AutoPET Control test datasets (Table 1). The SUV mean absolute error (SUV MAE), mean absolute relative error (MARE) and the fraction of explained variance were calculated for the whole body volume. A visual assessment was performed on two lymphoma test studies.

### Application of ntPET

#### Normal twin correction

Study attributes like patient sex, temperature, body fat mass, and tracer uptake time can significantly impact the SUV-values of certain organs^15,16^. This added variance can ultimately obscure or confound disease-related effects in the statistical analysis of organ SUV_mean_ values. However, since the ntPET image is optimized to account for exactly these predictably occurring normal effects, one may remove the effects from the SUV_mean_ by subtracting with the SUV_mean_ of the ntPET (ntSUV_mean_). We define this “regressing out” of normal effects as a “twin correction” (tc), and it is related to the variance-reducing CUPED technique used in statistical A/B testing^23^:

$$\:tcSU{V}_{mean}=SU{V}_{mean}-htSU{V}_{mean}+C$$

Here, *C* is the population-wide per-organ average htSUV_mean_ included solely for ease of plotting and interpretation. Whereas normalization techniques like lean body mass normalization are intended to correct for singular patient-specific effects, i.e., that of fat body mass, the twin correction can be interpreted as an all-in-one normalization to the patient’s anatomy, demographics and acquisition parameters^24^.

To assess whether the twin correction succeeds at this, we analyzed the Stable Lymphoma test split to estimate the variance reduction obtained by going from SUV_mean_ to tcSUV_mean_ for five selected organs: subcutaneous fat, visceral fat, skeletal muscle, liver, and aorta. The first three organs are the major tissue types available with TotalSegmentator while the liver and aorta were chosen due to their importance as reference regions in several diseases^2,25,26^.

For the same organs, we tested whether the following study attributes had a significant effect on both the SUV_mean_, tcSUV_mean_, and lean-body-mass normalized SUV_mean_ (SUL_mean_): age (> 68yrs/<68years), fat body mass (kg), sex (M/F), uptake time (> 63 min/<63 min) and season (winter/spring/summer/fall), which is a surrogate for temperature. Lean body mass was calculated using the James formula^27^. We assumed a linear relationship between fat body mass and SUV and tested the significance of the slope, and the remaining continuous covariates were binarized with respect to the median. The seasonal effect was quantified via ANOVA and all other effects via Welch t-tests.

#### Unsupervised anomaly detection

A simple patient-specific voxel-wise anomaly map was derived by taking the difference between PET and ntPET images and smoothing with a 4 mm gaussian filter to minimize the effect of noise and small misalignments. Tumor masks were predicted by thresholding the anomaly maps at a fixed value. Anomalies in the kidneys, heart, bladder, brain, and small and large intestines were ignored due to high variation of tracer uptake. The threshold value was determined on the full AutoPET cancer test by a greedy search that optimized the dice score. The segmentation performance was quantified in terms of dice score on the same AutoPET cancer using this threshold, leading to an optimistic best-achievable estimate of the true segmentation performance^14^.

For the classification of Stable and Active Lymphoma datasets, no ground truth tumor masks were available. Instead, the predicted tumor masks were used to calculate the per-study total lesion glycolysis (TLG), that is, the segmented tumor volume multiplied by its SUV_mean_^8^. We then used the TLG as a biomarker to classify stable and active test cases and reported the area under the receiver operator characteristic curve (AUC). For comparison, the dice and AUC scores were also reported for a fully supervised nnUNet model trained specifically to perform tumor segmentation on the AutoPET dataset^28^. Selected studies of the AutoPET and Stable Lymphoma cohort were visualized to qualitatively evaluate sources of false positive and false negative segmentations.

## Results

### Evaluation of the ntPET

**Fig. 2:**
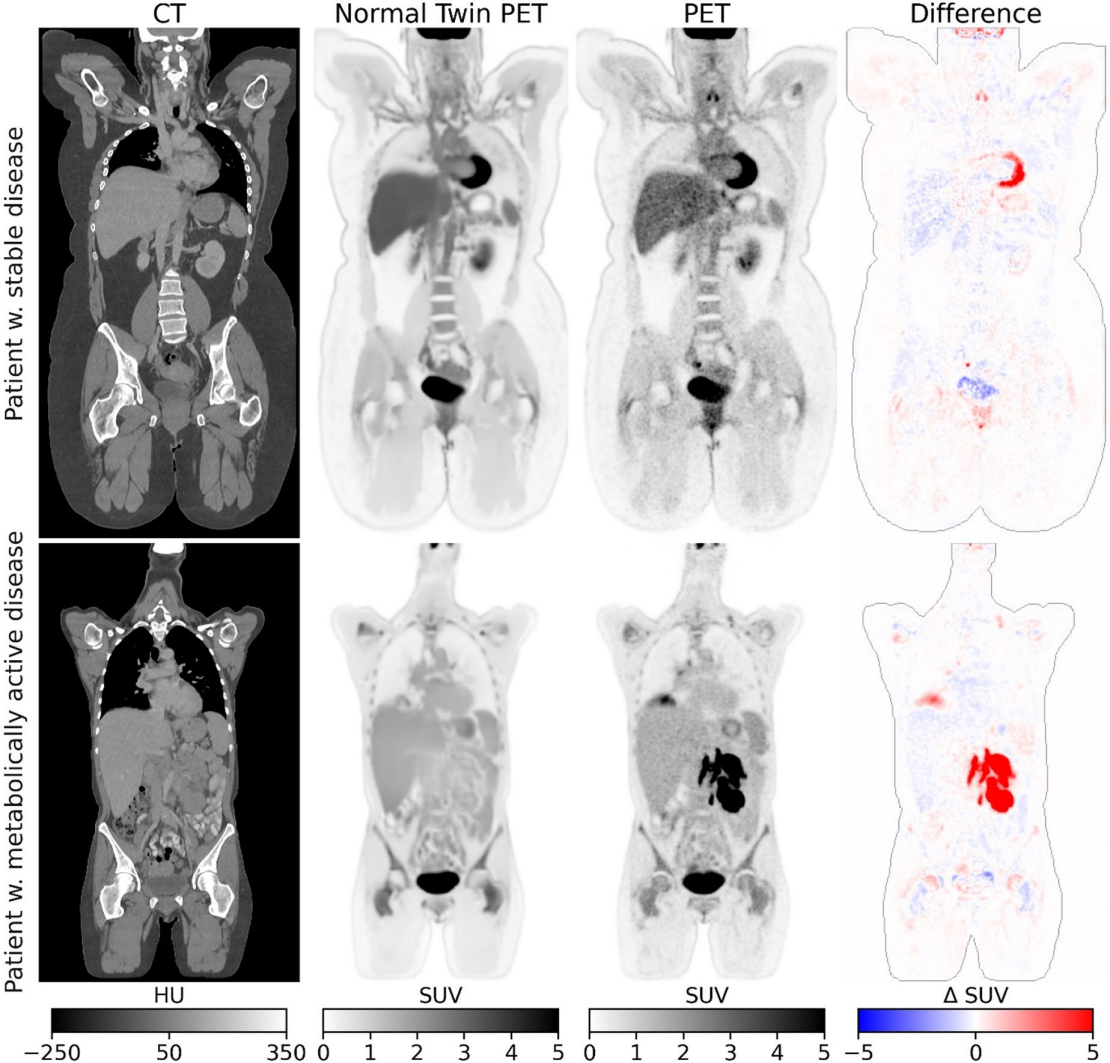
Two characteristic PET/CT test studies from the stable (top) and metabolically active (bottom) lymphoma cohorts, showing the corresponding normal twin PET images and the difference between true and normal twin PET images. Normal FDG variations are captured by the ntPET, resulting in near-zero differences in normal regions and large differences in malignant regions.

The synthesized ntPET images incrementally improved with the inclusion of study attributes, sCTs, and segmentations, with the best model obtaining an explained variance of 89.3%, a MARE of 18.0%, and an SUV MAE of 0.158 (Table 2). Notably, the ntPET images of the LAFOV stable lymphoma dataset scored better than those of the non-LAFOV AutoPET cohort, and all UNet-based models outperformed the constant organ map. Figure 2 shows the ntPET and ground truth PET images of a stable and an active lymphoma test patient. The ntPET in its current form conforms to the anatomy of the patient but performs less accurate in areas of high and varying uptake like the heart, brain, bladder, vocal cords, brown adipose tissue, kidneys, and ureters. Other sources of errors included sequelae after surgery and treatment induced elevated FDG-uptake in the bones. Note that the ntPET deviates significantly from the true PET in malignant regions, which is desired for later unsupervised anomaly detection. Supplementary Figure S2 demonstrates that disease masking is essential for this behavior, as models trained without masking learn to predict elevated uptake in CT-discernable tumors. The best performing *UNet CT + attr.+sCT + seg.* model was used for all subsequent analyses.

**Table 2:** ntPET model performance on stable test sets. Incremental improvements shown with the addition of study attributes (attr.), synthetic CT (sCT), and segmentations (seg.). Metrics: explained variance, SUV mean absolute error, and mean absolute relative error (MARE%).

	Lymphoma stable (*n* = 177)			AutoPET (*n* = 91)		
ntPET model \ metric	*Exp. Var*	*SUV MAE*	*MARE%*	*Exp. Var*	*SUV MAE*	*MARE %*
*Constant organ map*	52.9%	0.349	51.3%	50.8%	0.403	96.4%
*UNet CT*	75.1%	0.212	23.1%	67.4%	0.339	33.9%
*UNet CT + attr.*	75.3%	0.210	22.7%	67.1%	0.335	33.5%
*UNet CT + attr.+seg + sCT.*	89.3%	0.158	18.0%	87.9%	0.204	25.3%

### Normal twin correction

**Fig. 3:**
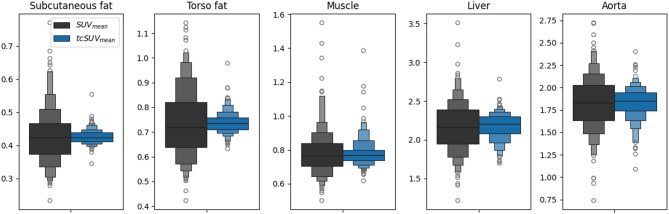
SUV distributions before (black) and after twin correction (blue). Variance reductions: subcutaneous fat (92%), visceral fat (90%), muscle (60%), liver (74%), aorta (65%).

**Fig. 4:**
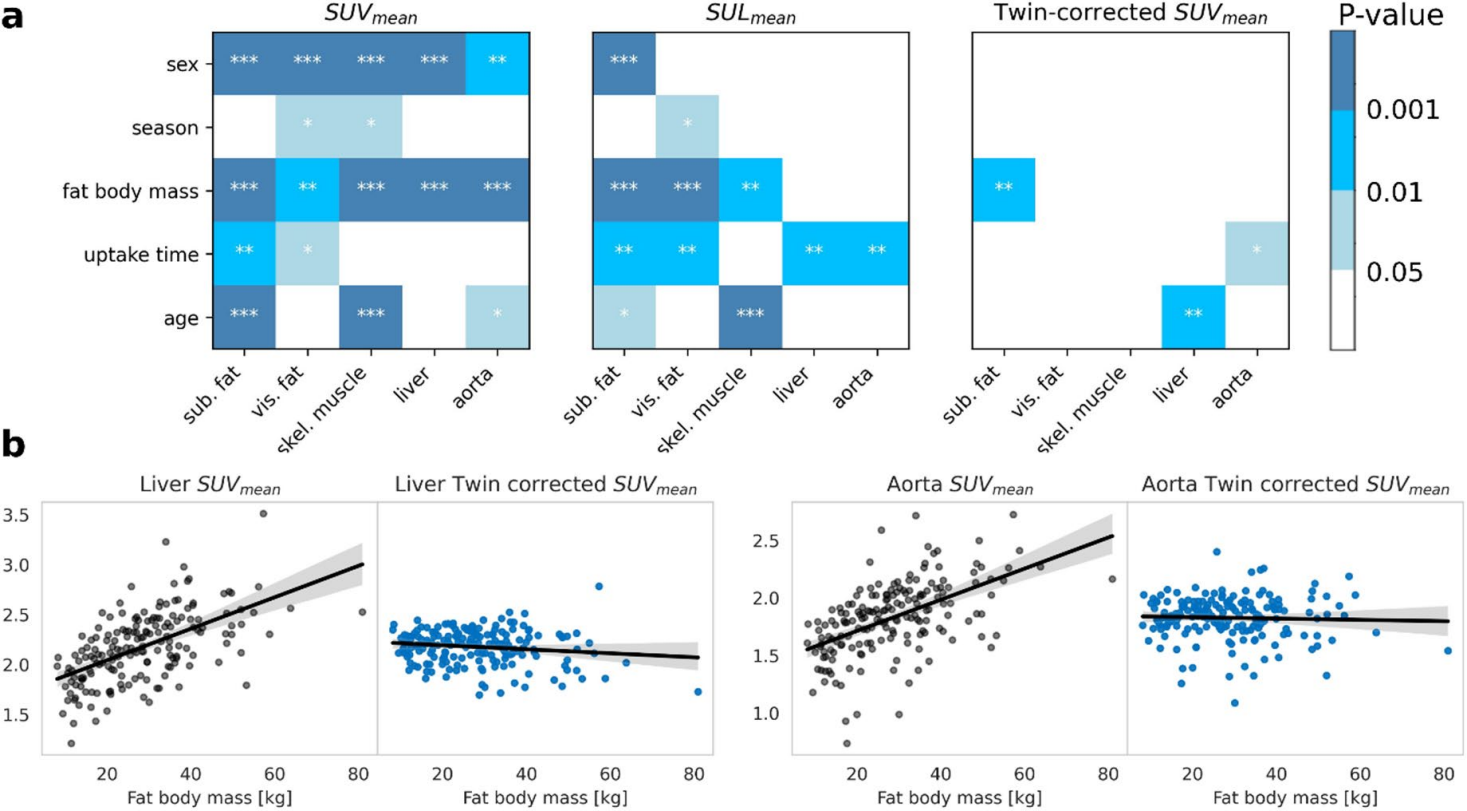
Twin correction removes patient-specific effects. (**A**) P-values showing reduced associations with patient attributes after correction. (**B**) Fat body mass effect on liver and aorta SUV reduced significantly by twin correction.

Compared to an ordinary SUV_mean_ analysis, the twin-corrected SUV_mean_ analysis exhibited significantly lower variance, particularly for subcutaneous fat (92% reduction) and visceral fat (90% reduction) (Fig. 3). This can likely be attributed to the tcSUV_mean_ accounting for the influence of patient-specific normal effects, as shown in the p-value table of Fig. 4. Consistent with literature, we observe statistically significant association between SUV_mean_ organ measurements and patient sex, age, fat body mass, study season, and uptake time^15,29^. While the lean body mass normalization accounts for the effect of fat body mass on the liver and aorta, the effects of uptake time and age remain significant. By contrast, the tcSUV_mean_ measurements show barely any statistically significant associations with the patient covariates, which indicates that the ntPET-based twin correction has successfully regressed out the patient-specific normal effects. As an example, Fig. 4 illustrates how the twin correction removes the linear effect of fat body mass on the liver and aorta SUV measurements.

### Unsupervised anomaly detection

**Fig. 5:**
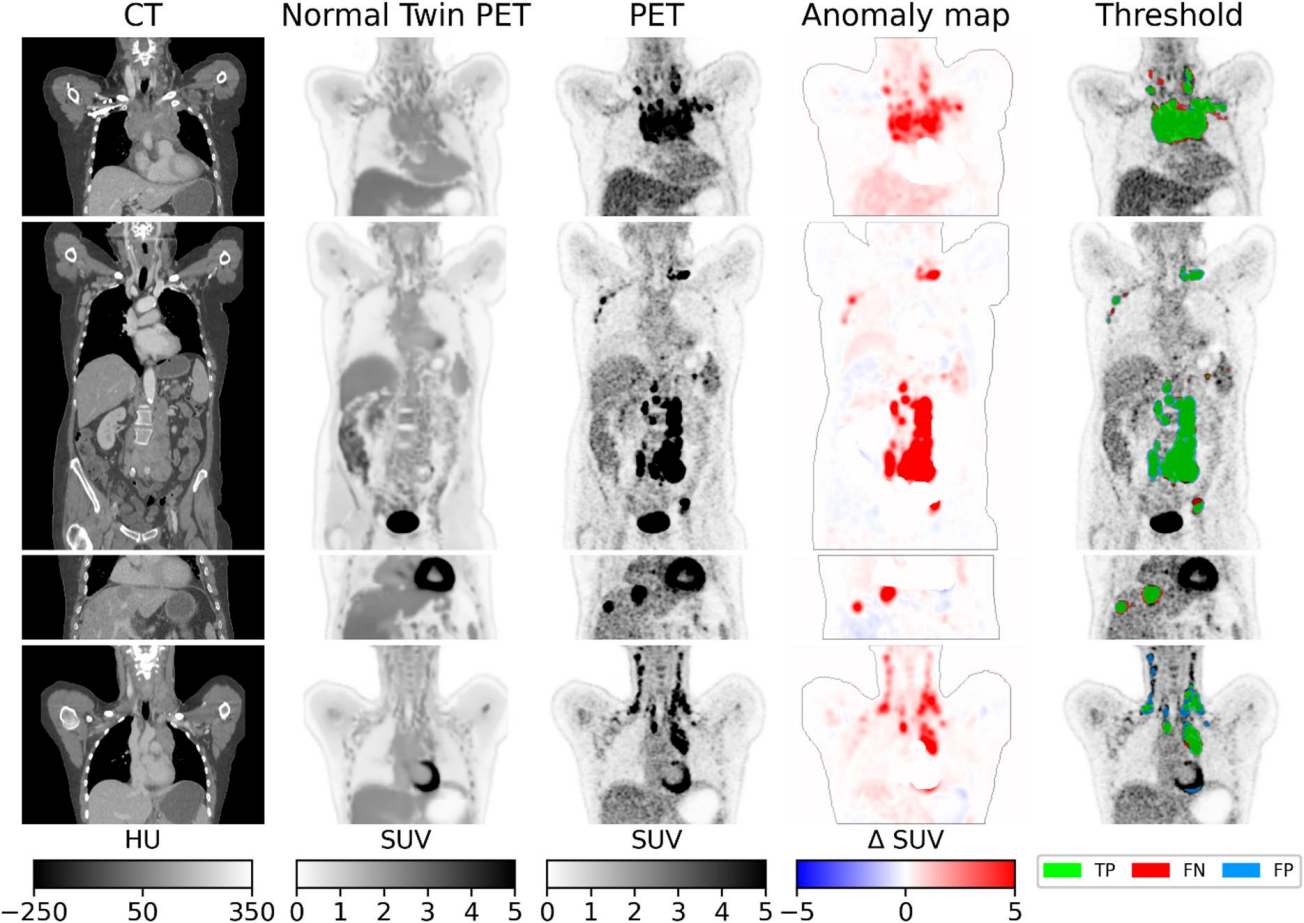
Twin-based tumor segmentation on four AutoPET test cases.

Anomaly maps were generated for the Active (*n* = 121) and Stable (*n* = 117) Lymphoma test studies, and for the 501 AutoPET Cancer studies. Tumor segmentation predictions were obtained by thresholding the anomaly maps at 1.8 SUV. This threshold value was obtained on the full AutoPET test cohort via a greedy search. Table 3 lists the average dice score of the investigated models on the AutoPET dataset and Fig. 5 depicts the segmentations of four randomly chosen patients. Note how the ntPET and true PET images differ significantly in areas of malignancy, which enables the automatic segmentation of the tumors. The ntPET model achieved a dice of 49.3%, which is better than the constant organ map model (39.3%), but worse than that of a fully supervised nn-UNet (65.1)%. However, the nnUNet model required ground truth tumor delineations for supervised training, while our unsupervised ntPET-based model did not.

**Table 3:** Performance of the normal twin PET images on two downstream tasks; tumor segmentation and separation of stable/active lymphoma cases. *Dice score represents 5-fold validation score (*n* = 501).

Model	AutoPET segmentation Dice Score (*n* = 501)	Lymphoma stable/active AUROC (*n* = 298)
(supervised) nnUNet*	65.1%	88.3%
(unsupervised) *Constant organ map*	39.3%	67.5%
(unsupervised) *ntPET*	49.3%	76.5%

Using the predicted tumor segmentations, TLG scores were obtained for the lymphoma test set and used to separate the active and stable test studies (Table 3). The ntPET model outperformed the constant organ map model, achieving an AUC of 76.5%. Although our ntPET-based UAD method identified abnormal regions in all active studies, the TLG-based classification was very sensitive to false positive segmentations of areas with reactive or physiological uptake as exemplified in Supplementary Figure S3. The supervised nn-UNet suffered significantly less from this issue and obtained a higher AUC of 88.3%.

## Discussion

This study presents a novel approach for generating whole-body normal twin PET images which conform to a patient’s unique anatomy. This was achieved by training a UNet on a large cohort of pseudo-normal PET/CT studies with disease suspicious areas manually delineated and excluded. The inclusion of patient demographics and acquisition parameters in the ntPET predictive model, allowed the capture and subsequent removal of normal effects from the SUV_mean_ measurements via a novel twin correction method. Finally, we showed the feasibility of using the ntPET images for unsupervised anomaly detection, achieving promising segmentation performance in the absence of ground truth segmentations.

The ntPET method successfully generates patient-specific PET images of predictable normal uptake patterns, maintaining normal appearance even in cancer patients (Figs. 2 and 5). Malignant regions show significantly lower [^18^F]FDG uptake in ntPET than in the true PET as desired and model performance improved incrementally with additional inputs (Table 2): sCT corrected PET/CT misalignment, while organ segmentations and demographics provided global context for patch-based predictions.

The relationship between study attributes and uptake was correctly learned by the model as evidenced by the reduced confounding effects on SUV_mean_ analyses after twin correction (Figs. 3 and 4). This finding highlights the potential of the twin correction as an all-in-one patient-specific normalization to anatomy, age, sex, and acquisition parameters. In particular, the removal of normal effects substantially reduced the variance of organ SUV_mean_ measurements, which could be valuable for future endeavors in expanding the diagnostic and prognostic potential of PET images. In contrast to the focal and [^18^F]FDG-avid lesions of malignant diseases, the pathology of non-malignant diseases may manifest as subtle and diffuse variations in the FDG-PET connectome^30^. Previously such subtle deviations may have been obscured by patient-specific normal effects, which we have shown can account for up to 90% of the SUV_mean_ variation (Fig. 3). By instead opting for the variance-reduced tcSUV_mean_ measurements, it may be possible to obtain more statistically significant separation of disease and control populations.

The unsupervised anomaly detection (UAD) approach utilizes the ntPET model to localize suspicious uptake without expert-derived delineations^9^. However, supervised segmentation models outperform UAD when ground truth labels are available, as shown in Table 3. The UAD’s lower performance occurs because deviations from the normal PET distributions don’t always signify pathology - they may result from physiological or reactive uptake in organs like vocal cords, lymph nodes, muscle, and bone marrow (Figs. 2 and 5). This suggests a single ntPET image may be insufficient to characterize the normal state, as patients could have multiple equally normal PET variants. To address this, ntPET could be modeled as a distribution using probabilistic machine learning. Future studies could enhance specificity by conditioning on additional clinical information (e.g., radiotherapy schemes) and developing advanced scoring methods to differentiate pathology from other anomalies. For instance, a secondary model could categorize deviations as physiological, reactive, or malignant.

Related work in UAD often adopt autoencoders such as variational autoencoders (VAE) or vector-quantified (VQ) autoencoders to model the distribution of normal PET images^14,31–33^. Since such architectures receive PET images as input, careful regularization of the latent space is necessary to prevent the model from learning an identity mapping shortcut^17,34^. Such a shortcut is detrimental to UAD since anomalies present in the input PET can carry through and persist in the predicted normal PET. Alternatively, atlas-based registration methods for whole-body PET normalization face significant challenges beyond the brain domain, including registration failures in anatomically variable regions (abdomen, thorax), unrealistic deformations when aligning patients with varying body habitus, errors at air-tissue interfaces, and requirements for standardized positioning including arm removal from the field of view^35,36^. In contrast, the ntPET approach avoids both the identity mapping problem and geometric registration constraints by conditioning on CT rather than PET: no latent space regularization is necessary, and normal references are synthesized directly in the patient’s native space, naturally accommodating anatomical variations without registration. The skip connections of the UNet also allow for high spatial fidelity, which is important for UAD of small lesions, and the training objective corresponds simply to maximizing the likelihood of the pseudo-normal PET images conditioned on the patients’ anatomy.

The ntPET approach offers several advantages over supervised classification and regression models for whole-body PET/CT analysis. Firstly, the interpretation of ntPET as a patient’s normal variant provides intuitive explainability that may improve clinical applicability^6,13^. In UAD, anomalies are simply interpreted as deviations from the normal state. When segmentations appear ambiguous or incorrect, clinicians can visually assess the ntPET to understand the source of deviation. This transparency contrasts with conventional classification and segmentation networks, which often lack interpretable outputs and provide little insight into their predictions^4,5^. Importantly, the ntPET can be automatically generated in ~ 1 min following PET/CT acquisition, enabling availability at the time of clinical reading. Secondly, the twin correction offers applicability for standardizing quantitative measurements across diverse scanners and protocols, with ntPET images serving as patient-specific reference baselines analogous to population atlases in brain PET.

Finally, since the ntPET methodology learns characteristics of normal PET images, it is inherently disease-agnostic, unlike segmentation and classification models trained for specific diseases. Notably, tumor segmentation and twin correction emerged as applications of the trained twin model without requiring specific finetuning for these tasks. This versatility suggests the ntPET approach could be adapted to various clinical routines involving whole-body [^18^F]FDG PET/CT. Other potential applications include serving as priors in image reconstruction, facilitating PET-to-PET registration, or detecting anomalies across different diseases^13^. This disease-agnostic approach could ultimately reduce dependence on specialized training datasets, which can be expensive to obtain and annotate^6,7^.

One major limitation of the ntPET approach is its reliance on non-control PET/CT data and the assumption that the MIP-based masking removes all clinically significant diseases. For instance, cancer patients often have non-malignant comorbidities, and including these in the training data may prevent the ntPET model from accurately estimating normal uptake. However, since collecting large PET/CT datasets of negative controls is costly, future work should improve methods to utilize existing clinical PET/CT studies and negative control datasets could then be reserved for finetuning and validation.

Another limitation stems from the use of sCTs as input to the generative model. If the PET image shows evidence of disease, there is a risk that this information might propagate to the predicted sCT and subsequently influence the generated ntPET. Since this could potentially lead to the presence of disease in the ntPET, alternative methods should be explored for correcting misalignment between PET and CT images. One possibility is to perform cross-modal nonlinear registration prior to training the generative model.

Addressing these limitations through probabilistic modeling, improved disease exclusion, and intrastudy registration will enhance ntPET applicability for personalized quantitative analysis. Further research should focus on validating the ntPET approach and exploring its integration with other study attributes to improve UAD and test new downstream applications.

## Conclusion

The proposed AI-based method successfully generated whole-body normal twin PET images that reflect a normal [^18^F]FDG distribution tailored to the patient’s anatomy, demographics, and acquisition parameters. In the absence of negative control studies for training, we used patient studies in combination with disease masking and showed that we were still able to obtain normal appearing ntPET images. The application of ntPET showed promise for unsupervised anomaly detection, and the novel twin correction method significantly reduced the impact of normal effects on SUV_mean_ organ measurements, which could aid connectome analyses and improve the diagnostic potential in non-malignant diseases. Future research should focus on probabilistic modeling, improved disease exclusion, and validation on negative controls.

## Supplementary Information

Below is the link to the electronic supplementary material.

Supplementary Material 1

## Data Availability

The retrospective datasets generated and analyzed during the current study are not publicly available nor available for sharing due to limitations of the Danish National Committee on Health Research Ethics approval. Please contact the corresponding author Christian Hinge at christian.hinge@regionh.dk for further information.
