# Supplementary material for: Normal twin PET: personalized generative modeling for confounder correction and anomaly detection in whole-body PET/CT
Source: Sci Rep. 2025 Nov 28;15:42662. doi: 10.1038/s41598-025-26827-y (PMC12663462; doi:10.1038/s41598-025-26827-y)
Supplement: Supplementary file 1 — Supplementary Material 1 [file 41598_2025_26827_MOESM1_ESM.docx]

## Supplementary Information


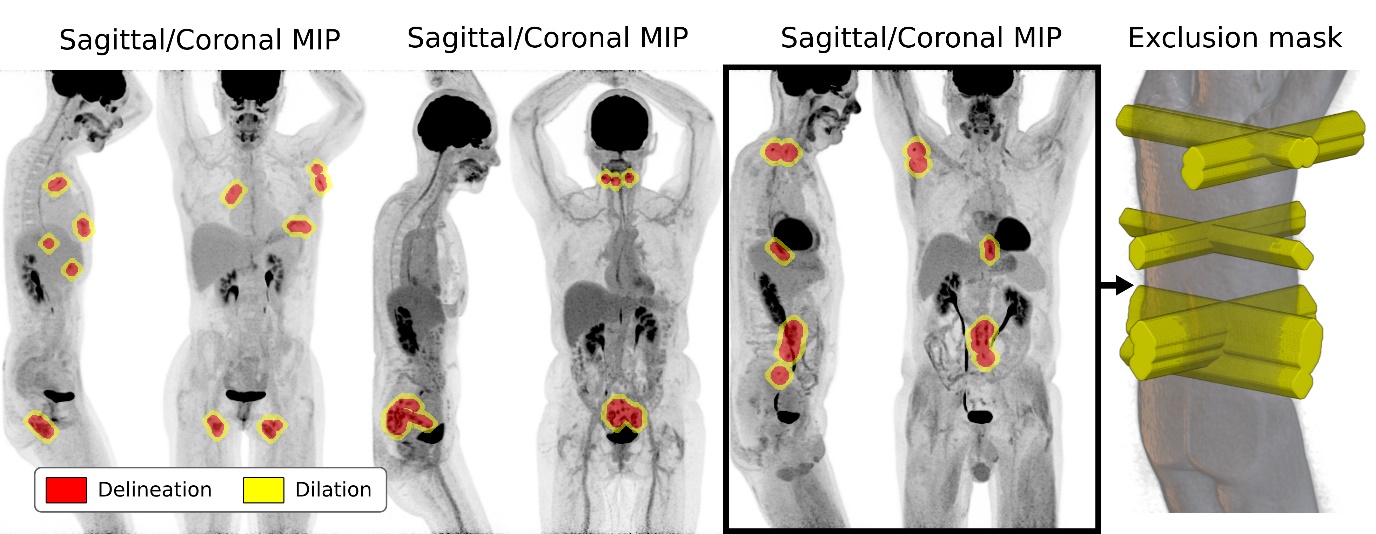


**Supplementary Fig. S1:** Disease masking procedure. Manual MIP segmentations (red) of suspicious areas were dilated (yellow) and expanded to create 3D exclusion masks for model training.


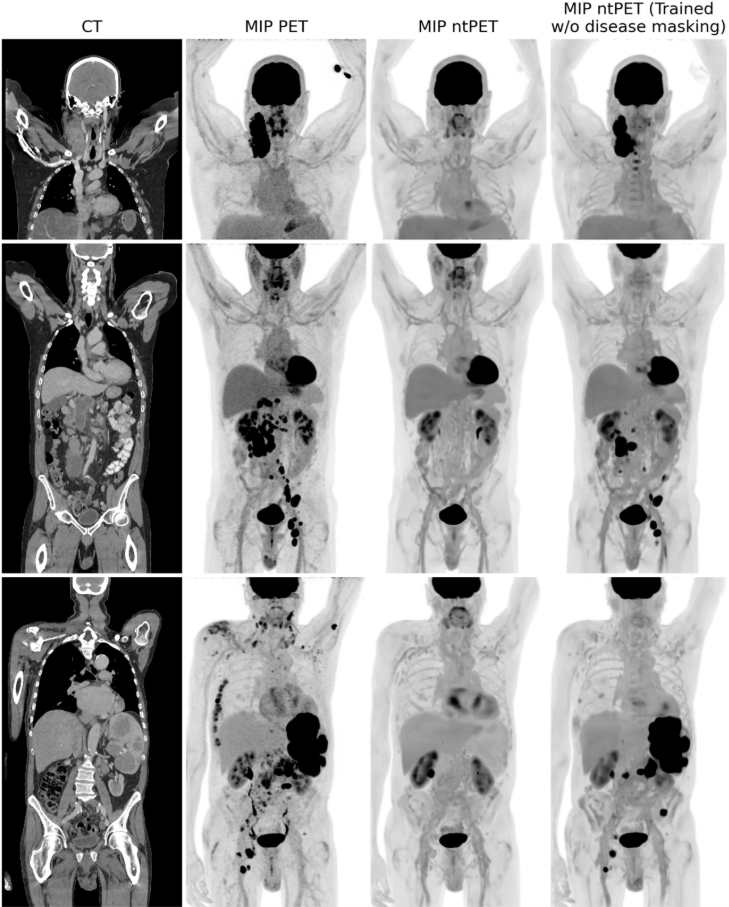


**Supplementary Fig. S2.** Disease masking prevents tumor uptake patterns from being learned as normal. Three patients from the Active Lymphoma cohort showing ntPET models trained with (third column) and without (fourth column) disease masking. Without disease masking, the model learns to predict elevated FDG uptake in malignant regions visible on CT, as these patterns are present in the training data. While this reflects the actual PET distribution, it is undesirable for generating "normal" reference images. Disease masking ensures the ntPET reflects normal tissue metabolism regardless of structural abnormalities in CT.


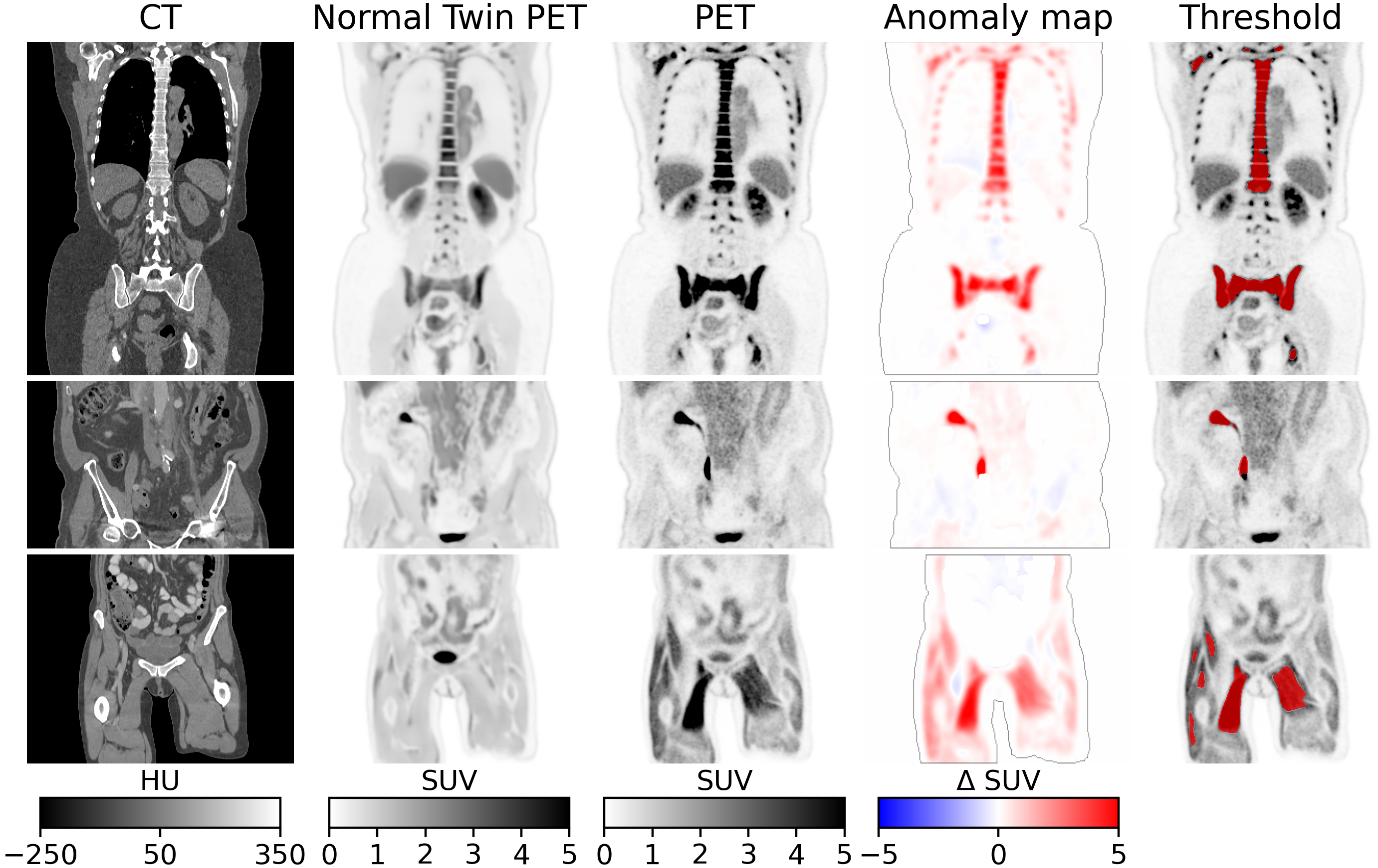
**Supplementary Fig. S3** Common false-positive patterns in twin-based tumor segmentation: treatment-related bone marrow uptake (top), excretion of FDG in the ureter (middle), and muscle activity (bottom).
